# Adapting Proteostasis and Autophagy for Controlling the Pathogenesis of Cystic Fibrosis Lung Disease

**DOI:** 10.3389/fphar.2019.00020

**Published:** 2019-02-01

**Authors:** Manish Bodas, Neeraj Vij

**Affiliations:** ^1^Department of Medicine, University of Oklahoma, Oklahoma City, OK, United States; ^2^Department of Pediatric Pulmonary Medicine, Johns Hopkins University School of Medicine, Baltimore, MD, United States; ^3^4Dx Limited, Los Angeles, CA, United States; ^4^VIJ Biotech LLC, Baltimore, MD, United States

**Keywords:** proteostasis, autophagy, cystic fibrosis, CFTR, ROS, protein-misfolding

## Abstract

Cystic fibrosis (CF), a fatal genetic disorder predominant in the Caucasian population, is caused by mutations in the cystic fibrosis transmembrane conductance regulator (*Cftr*) gene. The most common mutation is the deletion of phenylalanine from the position-508 (F508del-CFTR), resulting in a misfolded-CFTR protein, which is unable to fold, traffic and retain its plasma membrane (PM) localization. The resulting CFTR dysfunction, dysregulates variety of key cellular mechanisms such as chloride ion transport, airway surface liquid (ASL) homeostasis, mucociliary-clearance, inflammatory-oxidative signaling, and proteostasis that includes ubiquitin-proteasome system (UPS) and autophagy. A collective dysregulation of these key homoeostatic mechanisms contributes to the development of chronic obstructive cystic fibrosis lung disease, instead of the classical belief focused exclusively on ion-transport defect. Hence, therapeutic intervention(s) aimed at rescuing chronic CF lung disease needs to correct underlying defect that mediates homeostatic dysfunctions and not just chloride ion transport. Since targeting all the myriad defects individually could be quite challenging, it will be prudent to identify a process which controls almost all disease-promoting processes in the CF airways including underlying CFTR dysfunction. There is emerging experimental and clinical evidence that supports the notion that impaired cellular proteostasis and autophagy plays a central role in regulating pathogenesis of chronic CF lung disease. Thus, correcting the underlying proteostasis and autophagy defect in controlling CF pulmonary disease, primarily *via* correcting the protein processing defect of F508del-CFTR protein has emerged as a novel intervention strategy. Hence, we discuss here both the rationale and significant therapeutic utility of emerging proteostasis and autophagy modulating drugs/compounds in controlling chronic CF lung disease, where targeted delivery is a critical factor-influencing efficacy.

## Introduction

Cystic fibrosis (CF) is one of the most common fatal autosomal recessive disorders (Zhang et al., 2018), with emerging treatment options that have prolonged survival but limited success in diminishing overall mortality. Specially, subjects with homozygous F508-del (phenylalanine-508) mutation that suffer from the most serious form of ailment, lack effective treatment options that can warrant cure or normal survival. Briefly, chronic airway disease is a major contributor of the morbidity and mortality in CF subjects (Cantin et al., 2015; Mall, 2016), and accessibility of several novel and more potent therapeutic options has allowed substantially improved survival. Majority of these therapeutic strategies are aimed at controlling symptomatic CF-lung disease, while some of the newer strategies are designed to target the primary or “root” cause of the disease, which is the mutation(s) in the cystic fibrosis transmembrane conductance regulator (*Cftr*) gene (Esposito et al., 2016; Hudock and Clancy, 2017; Maiuri et al., 2017; Zhang et al., 2018). There are about 1700 known mutations affecting the generation (Class I), structure (Class II) or channel function (Class III–V) of the CFTR protein, and about 88% of these comprise of mutations that cause the protein misfolding defects (Class II) (C.F. Foundation, 2018). People with these mutations on both the copies of their *Cftr* gene demonstrate the classical manifestations of CF lung disease, such as a thick and sticky mucus, mucin hypersecretion, elevated inflammatory-oxidative stress and/or persistent bacterial infections, which collectively result in severe airway obstruction (Bodas and Vij, 2010; Bodas et al., 2018a). Evidence from newer animal models of CF and some clinical data indicate that symptoms of lung disease are present at very early age, or even at birth, thus proposing the concept of congenital origin of CF lung disease (Stoltz et al., 2015). With progressing age, persistent exacerbations primarily caused by *Pseudomonas aeruginosa* (*Pa*) and the ensuing IL-8 mediated persistent neutrophilic inflammation are the hallmark of clinical CF lung disease and a major contributor to irreversible lung damage as well as CF-related fatalities (Bodas and Vij, 2010; Ferrari et al., 2017; Bodas et al., 2018a).

**FIGURE 1:**
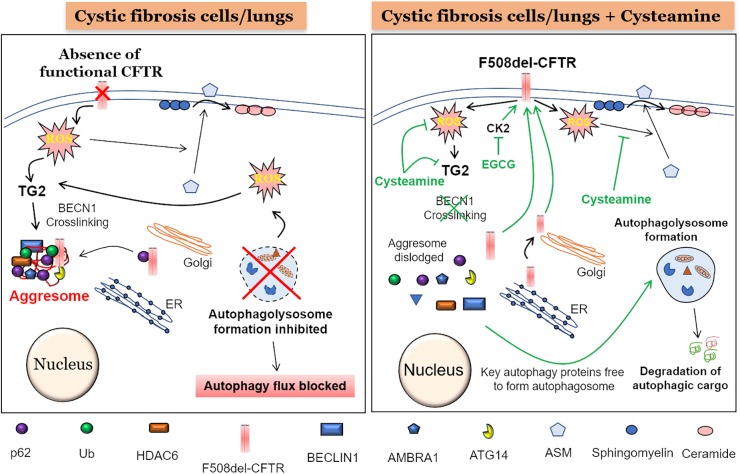
Mechanism of cysteamine mediated autophagy induction and F508del-CFTR rescue. (Left panel) The absence of a functional CFTR at the plasma membrane (PM) leads to elevated reactive oxygen species (ROS) levels which cause activation of transglutaminase-2 (TG2). An active TG2 mediates cross-linking of crucial autophagy proteins such as Beclin1 (BECN1), ATG14, and AMBRA1 into Ub+/p62+/HDAC6+ aggresome bodies, render BECN1 and other autophagy proteins unavailable for the formation of autophagosome and thus blocking the subsequent autophagy flux process. Moreover, an accumulation of p62 could lead to aggresome sequestration of newly synthesized F508-delCFTR, thereby preventing its PM translocation. Additionally, accumulation of damaged mitochondria leads to more ROS production, further promoting TG2-mediated BECN1 crosslinking and autophagy inhibition. Further, the ROS mediated translocation of acid sphingomyelinase (ASM) from cytoplasm to PM, leads to increased conversion of sphingomyelin to ceramide, which is a deleterious sphingolipid implicated in CF pathogenesis. (Right Panel) The treatment of CF cells or mice with the autophagy inducing antioxidant drug, cysteamine, which is also an inhibitor of TG2, leads to prevention of BECN1 crosslinking. This results in dislodging of aggresome components resulting in availability of BECN1 and other key autophagy proteins to form the autophagosome, thus allowing the autophagy process to function and leading to the clearance of autophagic cargo. Moreover, decreased p62 levels due to a functional autophagy flux will possibly allow the newly synthesized F508-delCFTR to reach the PM and restore partial CFTR function, even though some of previously aggresome sequestered F508-delCFTR may be degraded by the active autophagy process. In addition to cysteamine, treatment with epigallocatechin-gallate (EGCG) at the time of cysteamine removal, potentiates the long-term stability of the PM-rescued F508-delCFTR, due to its inhibitory effect on protein kinase CK2, which would otherwise promote peripheral/PM degradation of F508-delCFTR. Additionally, a functional autophagy process means that the toxic aggregated proteins and other damaged organelles such as mitochondria are homeostatically degraded, thus decreasing overall ROS levels. Finally, cysteamine blocks the translocation of ASM from cytoplasm to the PM, thus reducing the conversion of sphingosine to ceramide, and preventing ceramide-mediated inflammatory-apoptotic signaling in CF cells and/or lungs.

In general, all the cellular processes that work to maintain a robust protein repertoire in the cell are collectively called the proteostasis network (PN) (Klaips et al., 2018). This complex molecular system tightly regulates the fate of a protein inside the cell, starting from its synthesis, folding, and maintenance of the folded functional state, to transport (trafficking), and eventual degradation. A vast amount of cellular energy is utilized to maintain the protein degradation machinery to get rid of misfolded, damaged, non-functional or even functional proteins that are no longer required by the cells. The main protein degradation or cellular clearance mechanisms include the ubiquitin-proteasome system (UPS) and the “autophagy-lysosomal pathway” (Korovila et al., 2017). The primary difference between proteasome and autophagy mechanisms is the type of cargo they can process as their starting material. The proteasome can only process proteins, while large protein aggregates, lipids and even damaged organelles can be processed and degraded by the autophagy pathway. Recent evidence suggests a strong inter-relationship between the proteasome and autophagy pathways (Bustamante et al., 2018) and thus it is not surprising that any disturbance in any of these mechanisms can form the basis of accumulation of aberrant proteins eventually leading to severe pathological conditions such as those seen in aging-related neurodegenerative diseases (Daniele et al., 2018; Klaips et al., 2018), proteinopathies (Chaudhuri and Paul, 2006; Hidvegi et al., 2015; Hartl, 2017), and genetic or environmentally induced chronic respiratory diseases such as CF (Lukacs and Verkman, 2012; Fraser-Pitt and O’Neil, 2015) and COPD (Tran et al., 2015; Bodas and Vij, 2017; Bodas et al., 2017; Vij, 2017), respectively. The deletion of phenylalanine-508 (F508-del) is the most common (about 80%) *Cftr* gene mutation associated with CF, which results in a misfolded CFTR protein that is unable to reach the plasma membrane (PM) (Lukacs et al., 1993; De Stefano et al., 2014). This results in the absence of mature CFTR ion-channel on the PM, leading to CFTR dysfunction, classically described as a chloride ion transport defect (Welsh et al., 1993). In addition, there is substantial evidence supporting the critical role of membrane-resident CFTR in regulating innate and adaptive immune responses in CF (Teichgraber et al., 2008; Vij et al., 2009; Bodas and Vij, 2010; Grassme et al., 2017; Svedin et al., 2017). Furthermore, a burgeoning number of studies now ascertain the crucial role of mature CFTR in regulating important cellular homeostatic processes such as proteostasis and autophagy, with a common consensus that autophagy is potentially inherently defective in CF (Gomes-Alves et al., 2010; Luciani et al., 2010, 2011; Bodas et al., 2012; Valle and Vij, 2012; Villella et al., 2013a). The genesis of defective autophagy in CF seems to be an inherent defect, as primary CF cells have diminished levels of several autophagy proteins (Abdulrahman et al., 2011, 2013), although the precise mechanism(s) are still unclear. Some interesting studies indicate the possible contribution of micro RNA’s (Tazi et al., 2016) and DNA methylation (Tazi and Amer, 2015), as both could regulate the expression of autophagy proteins in CF cells. Nonetheless, it is well documented that the absence of membrane CFTR leads to ROS-mediated SUMOylation of transglutaminase 2 (TG2), which prevents its ubiquitination and subsequent degradation by the proteasome, leading to its intracellular accumulation. This results in the crosslinking of Beclin-1 (BECN1), an important protein required for autophagosome formation, leading to defective autophagy, and accumulation of SQSTM1 (p62) (Luciani et al., 2010; Bodas et al., 2017), which favors aggregation of BECN1 and other autophagy related proteins into p62+HDAC6+ aggresome bodies (Figure 1). The misfolded F508del-CFTR is also sequestered into aggresome bodies, as the accumulation of p62 leads to inhibition of both protein (proteasome) and aggresome clearance. This aggresome trapping of F508del-CFTR prevents its proper trafficking to the PM that contributes to the initiation and progression of inflammatory-oxidative stress responses in the CF lungs (Luciani et al., 2010).

This suggests that future strategies for managing and treating CF will need to be focused on correcting the underlying proteostasis/autophagy impairment, mainly *via* rescuing F508del-CFTR to the PM, which would simultaneously control the inflammatory-oxidative stress response in the airways of CF subjects. Lately, proteostasis-modulators and autophagy-inducers have shown encouraging results in pre-clinical studies in correcting both the F508del-CFTR trafficking to the PM (CFTR-corrector) (De Stefano et al., 2014; Tosco et al., 2016; Vu et al., 2017; Hutt et al., 2018; Stincardini et al., 2018; Zhang et al., 2018), as well as dampening the inflammatory-oxidative stress responses (anti-oxidant/anti-inflammatory) (Romani et al., 2017; Stincardini et al., 2018), although these strategies were not very efficient in restoring other rare class II CFTR mutations (Awatade et al., 2018). Moreover, it is not known if rare CFTR mutations also lead to a autophagy defect, and thus many other CFTR modulators have been clinically tested to evaluate their efficacy in restoring the PM stability and function of different CFTR mutants (Lopes-Pacheco, 2016). This strategy is currently being developed as a personalized CF management plan and holds potential for CF patients with all classes of CFTR mutations (Lopes-Pacheco, 2016; Paranjape and Mogayzel, 2018). As an example, for people with the G551D mutation, the orally bioavailable potentiator drug, VX770, shows substantial promise as an inducer of mutant CFTR channel activity, and thus is FDA approved for clinical application in CF patients (Accurso et al., 2010). However, since about 80% of CF patients worldwide possesses the F508del-CFTR defect (Lopes-Pacheco, 2016), drugs that correct its PM trafficking, stability, and function have emerged as promising therapeutic pipeline for clinical validation in CF subjects. Since the single corrector drugs showed minimal clinical benefit, successful efforts have been made to develop combinatorial therapy for CF. In fact, several CFTR-corrector compounds have been clinically evaluated and few have even reached the CF patients in combination with CFTR-potentiator drugs Orkambi^®^, Symdeko^®^ (Birault et al., 2013; Wainwright et al., 2015; Mayer, 2016), although their present costs are humongous (Mayer, 2016; Bulloch et al., 2017). Additionally, a recent study tested a triple combination of pharmacological chaperones (VX809+MCG1516A+RDR1) and demonstrated better CFTR functional correction than VX809 alone (Carlile et al., 2018), thus providing potentially promising future therapeutics for CF subjects. Similarly, another triple combination therapy was tested using VX-659-tezacaftor-ivacaftor (Davies et al., 2018), and is currently in Phase III clinical trials (Sala and Jain, 2018).

Another important aspect of current CF drugs is the challenge of drug-delivery and/or *in vivo* bioavailability due to the notoriously thick and sticky mucus layer (Brockman et al., 2017; Vij, 2017). Considerable pre-clinical research is ongoing to address these crucial issues, and there has been some success in designing novel drug-delivery systems that achieve targeted drug-delivery and long-term bioavailability in the CF lungs (Vij et al., 2010; Vij, 2011, 2017; van Rijt et al., 2014). Although scientists and clinicians have come a long way on significantly improving the median survival age of CF patients to reach adulthood (∼40–50 years) (West and Flume, 2018), the disease is still incurable and numerous precious lives are lost at a very early age. Thus, continued basic and translational research is essential to develop a better armamentarium of preventive/therapeutic strategies to further improve patient survival and possibly find a cure to this life-restricting and life-taking genetic disease. The current perspective compiles some recent studies that have the potential to translate into emerging therapeutic strategies for CF subjects, with the focus on drugs/compounds that correct the underlying disease-promoting defect in proteostasis and autophagy, including the protein-processing defect in F508del-CFTR protein.

## Autophagy Inducers as Emerging CF Therapeutics

One of the foremost cellular proteostatic mechanism that regulates protein-processing is the catabolic autophagy process. There is a plethora of evidence in recent studies that a partial (in chronic obstructive pulmonary disease, COPD) (Cantin, 2016; Bodas et al., 2017; Vij, 2017; Shi et al., 2018) or complete loss (in CF) (Luciani et al., 2010; Cantin, 2016) of functional CFTR protein from the PM leads to reactive oxygen species (ROS)-mediated autophagy impairment. This results in the accumulation of misfolded CFTR in perinuclear aggresome bodies, which eventually promotes the development of the lung disease. In CF, the accumulation of misfolded F508del-CFTR leads to ROS mediated autophagy impairment that results in increased inflammatory-oxidative stress (Luciani et al., 2010) in the CF airways. Moreover, a non-functional CFTR also contributes to defective bacterial uptake, killing, and clearance (Brockman et al., 2017; Ferrari et al., 2017; Pehote et al., 2017; Shrestha et al., 2017), which contributes to persistent exacerbations and inflammation, eventually resulting in irreversible damage of the pulmonary architecture. Lately, some pre-clinical and clinical trials demonstrate the utility of autophagy inducing therapeutic compounds in controlling pathogenesis and progression of CF lung disease (Villella et al., 2013b; Junkins et al., 2014; Esposito et al., 2016; Romani et al., 2017; Stincardini et al., 2018), that have shown promise in Phase-I/II trials but none have hitherto reached the bedside yet (De Stefano et al., 2014; Devereux et al., 2015, 2016). As discussed before (Yang et al., 2013; Vu et al., 2017), some other autophagy inducing drugs are also tested as potential CF drug candidates but these may not reach the patients due to the high doses required for the treatment as well as an evidence of many off-target side effects (Yang et al., 2013; Vu et al., 2017). Thus, we highlight here the leading proteostasis/autophagy-modulating compounds/drugs, which can allow bedside translation as emerging CF drug candidates.

## Cysteamine: A Multi-Pronged Drug for CF

Cysteamine is an FDA-approved drug for nephropathic cystinosis and has been very effectively used for over 25 years (Besouw et al., 2013; Fraser-Pitt et al., 2018). Chemically, cysteamine is an endogenously present, water soluble aminothiol, generated as a consequence of coenzyme A metabolism (Fraser-Pitt et al., 2018). It is commercially available as Cystagon^®^ and Procysbi^®^ and can be administered orally, although with well documented side effects (Cherqui, 2012; Veys et al., 2016). Over the years, cysteamine was introduced as a potential CF drug and henceforth several studies have been conducted to evaluate its efficacy in controlling CF lung disease (Besouw et al., 2013; Charrier et al., 2014; Devereux et al., 2015, 2016; Brockman et al., 2017; Shrestha et al., 2017). Mechanistically, cysteamine is a TG2 inhibitor, which dislodges the aggresome assembly, which is sequestering key autophagy proteins and F508del-CFTR, thereby restoring autophagy and decreasing p62 levels (Figure 1). This allows forward trafficking of misfolded F508del-CFTR to the Golgi and PM, thus reinstating its chloride channel function. Moreover, decreased p62 levels might also prevent sequestration of newly synthesized aggregation-prone F508del-CFTR protein (Luciani et al., 2010) into aggresome bodies, thus allowing its trafficking towards the PM. Additionally, knockdown of p62 also mimics the F508del-CFTR rescuing effect of cysteamine, thus confirming the crucial role of p62 in regulating the levels of F508del-CFTR on the PM. Recently, we demonstrated that cysteamine blocked translocation of acid sphingomyelinase (ASM) enzyme to the PM, thus blocking the conversion of sphingomyelin to ceramide, a pathogenic bioactive lipid implicated in CF lung disease (Figure 1; Bodas et al., 2018b). This study adds another novel mechanism of cysteamine action in controlling inflammatory-apoptotic signaling in CF lung disease, although further pre-clinical and clinical studies are warranted to verify these mechanisms. Nonetheless, it is encouraging that cysteamine is being developed as a delayed-release capsule form (Lynovex^®^) (Charrier et al., 2014) and has undergone preliminary clinical studies in CF subjects. A relatively recent small single arm, phase 1/2a open label study was conducted to evaluate the tolerance and pharmacokinetics of cysteamine in CF patients (Devereux et al., 2016). The results indicated that although some adverse reactions were observed in CF patients who were given oral cysteamine, these were similar to the side effects seen in cystinosis subjects (Devereux et al., 2016). Overall, cysteamine was well tolerated and entered the bronchial secretions at concentrations higher than plasma (Devereux et al., 2016). In addition, a recent promising study in mice and human CF subjects was conducted using cysteamine and epigallocatechin-gallate (EGCG) as a combinatorial drug strategy (Tosco et al., 2016). The beneficial effects of this approach were attributed to autophagy-induction mediated restoration of F508del-CFTR to the PM by cysteamine, followed by enhanced stability of the PM-resident CFTR protein via inhibition of protein kinase CK2, by EGCG. Intriguingly, we and others recently described that cysteamine can be utilized as a multi-pronged CF-drug candidate, as its abilities are not restricted to just correcting the CFTR-dependent chloride ion transport defect. In fact, cysteamine possesses a diverse repertoire of beneficial properties such as anti-oxidant (Bodas et al., 2016, 2018b; Govindaraju et al., 2017; Vij et al., 2018), anti-inflammatory (Ferrari et al., 2017), autophagy-inducer (Esposito et al., 2016; Tosco et al., 2016; Ferrari et al., 2017), bactericidal (Charrier et al., 2014; Ferrari et al., 2017; Shrestha et al., 2017), mucolytic and anti-biofilm (Charrier et al., 2014; Brockman et al., 2017), which are all necessary to control acute or recurring exacerbations and CF pulmonary disease progression. Even though cysteamine is a strong CF-drug candidate, it’s utility is possibly restricted to patients with only the F508del-CFTR mutation, as it was not very effective in other types of CFTR mutations such as R560S-CFTR (Awatade et al., 2018) that warrants further evaluation. Moreover, in spite of all the beneficial properties, the main caveats in the use of cysteamine is its poor bioavailability and the requirement of a high dose which is difficult to achieve *in vivo* (Vu et al., 2017). Thus, novel attempts have been made to improve the bioavailability of cysteamine, as well as to decrease the effective dose, such as by conjugating it with a [3-fatty acid (docosahexaenoic acid, DHA)], which also has its own autophagy inducing properties via the AMPK pathway (Vu et al., 2017). This conjugate could effectively rescue F508del-CFTR to the PM at a substantially lower concentration, thus warranting its further evaluation in a clinical setting. In another report, nine “prodrugs” of -glutamyl-cysteamine were tested in cultured kidney cells, to overcome its major disadvantages (Frost et al., 2016). These prodrugs could undertake successful delivery of cysteamine into kidney epithelial cells with improved bioavailability and low toxicity (Frost et al., 2016). This approach seems promising and needs further evaluation in pre-clinical CF models.

We have previously demonstrated the utility of nanotechnology in the development of novel drug delivery systems aimed at sustained and targeted delivery to the CF airways (Vij et al., 2010; Vij, 2011, 2017; Brockman et al., 2017). Using a similar approach, we recently proposed the application of dendrimer technology in designing a novel drug-delivery system to improve cysteamine’s bioavailability and specificity. We developed a dendrimer-cysteamine conjugate formulation (PAMAM-DEN^CYS^), and tested its ability to induce trafficking of F508del-CFTR to the PM in CF cells (Brockman et al., 2017). Although this was an pre-clinical early stage investigation, we were able to demonstrate key therapeutic signatures such as rescue of F508del-CFTR from the aggresome bodies and it’s trafficking to the PM, as well as control of *Pa* infection and growth, and mucolytic potential (Brockman et al., 2017). Therefore, this novel PAMAM-DEN^CYS^ conjugate has a potential for further development as an emerging CF therapeutic strategy, as it corrects the proteostasis and autophagy impairment, which is the central disease-promoting mechanism in pathogenesis of chronic CF lung disease.

## GSNO and GSNOR Inhibitors

**FIGURE 2:**
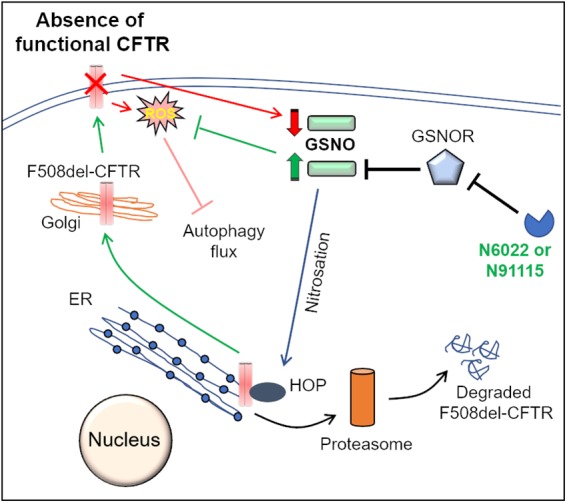
Mechanisms of GSNOR-inhibitors mediated rescue of F508del-CFTR to the PM. Molecular chaperones such as Hsp70Hsp90 organizing protein (HOP) regulate CFTR biogenesis and proper trafficking to the PM. In the ER, association of HOP with F508del-CFTR leads to its degradation via the ER-associated proteasomal pathway. S-nitrosoglutathione (GSNO), a cellular nitric oxide (NO) donor, modulates the function of HOP by its S-nitrosation. In the absence of a functional CFTR, cellular GSNO levels are low, which results in decreased S-nitrosation of HOP, which promotes proteasome mediated degradation of F508del-CFTR. The cellular levels of GSNO are tightly regulated by the enzyme, GSNO-reductase (GSNOR), which mediates the catabolism of GSNO. Pharmacological inhibition of GSNOR using N6022 or N91115 increases GSNO levels that leads to increased S-nitrosation of HOP. It is believed that an increase in HOP S-nitrosation hampers its association with F508del-CFTR, thereby allowing the forward trafficking and maturation of F508del-CFTR. Moreover, recent studies from our group also indicate that GSNO augmentation using N6022 can control the elevated ROS levels and thus correct the ROS-mediated autophagy flux impairment in CF.

Another interesting strategy to correct the proteostasis and autophagy defect in CF is through nitric oxide (NO)-augmentation, which facilitates the rescue of misfolded F508del-CFTR protein to the PM. Some previous reports propose the use of NO-donors (such as S-nitrosoglutathione, GSNO) or the inhibitors of GSNO-reductase (GSNOR), in controlling airway inflammation in experimental allergic asthma (Blonder et al., 2014) and CF models (Zaman et al., 2006, 2014, 2016; Rafeeq and Murad, 2017). In the lungs, NO and its reservoir, GSNO, play a very crucial role in the maintaining airway smooth muscle tone and controlling inflammation (Que et al., 2009; Sun et al., 2011). The levels of GSNO are tightly regulated by GSNOR, the enzyme which degrades GSNO (Sun et al., 2011). In fact, GSNO levels are diminished, with a concomitant increase in GSNOR levels, in both asthmatic and CF lungs (Que et al., 2009; Sun et al., 2011; Zaman et al., 2016), indicating that altered NO signaling contributes to asthma and CF pathogenesis. Mechanistically, the GSNO-mediated *S*-nitrosylation and subsequent degradation of Hsp70/Hsp90 organizing protein (HOP) favors the forward trafficking of CFTR to the PM (Figure 2; Odunuga et al., 2004; Marozkina et al., 2010). The findings that GSNO could increase the expression, maturation and function of both WT and F508del-CFTR in human bronchial epithelial cells led to clinical testing of a GSNOR-inhibitor, N91115 (Cavosonstat, Nivalis Therapeutics) (C.F. Foundation, 2015; Donaldson et al., 2017). The study reported that N91115 was well tolerated over a 28 day period, with no dose-limiting toxicities and no safety issues (Donaldson et al., 2017), albeit the study was discontinued in the Phase 2 stage as no improvement in lung function was observed in CF subjects. We recently reported that apart from its CFTR rescuing property, GSNO augmentation by using either GSNO or a GSNOR-inhibitor (N6022) effectively diminished CS-induced inflammatory-oxidative stress and also corrected the autophagy impairment (Bodas et al., 2017), thus targeting the underlying cause of CFTR dysfunction and resulting CF lung disease pathogenesis and progression. In fact, N6022 has been tested in clinical trials on CF patients with somewhat encouraging outcomes (C.F. Foundation, 2014; Rafeeq and Murad, 2017). The autophagy inducing property of GSNO or N6022 could be attributed to its rescue of CFTR to the PM (Zaman et al., 2016; Bodas et al., 2017), or other mechanisms such as its inhibitory effect on mTOR (Montagna et al., 2016), or its anti-oxidant function (Rauhala et al., 1998; Khan et al., 2011; Bodas et al., 2017). The *in vivo* application of N6022 could be restricted because of its low bioavailability, due to the presence of the highly polar imidazole group (Sun et al., 2011). Thus, GSNO-augmentation has the potential to be further tested in CF, where modifications in dosing and concurrent development of airway-delivery methodology can allow successful clinical outcomes.

## Potential Application of Fisetin as a Nutraceutical for CF

A plant derived nutraceutical, Fisetin (3,3′,4′,7-tetra hydroxyflavone), demonstrates the potential to be a future CF drug candidate (Pal et al., 2016). Ongoing, experimental and clinical research is investigating the preventive and therapeutic properties of Fisetin in chronic inflammatory conditions (Pal et al., 2016), neurological diseases and various types of cancers (Pal et al., 2016). Previous studies have described Fisetin as a potent anti-oxidant (Khan et al., 2013; Pal et al., 2016; Govindaraju et al., 2017), anti-inflammatory (Khan et al., 2013; Pal et al., 2016), bactericidal (Pehote et al., 2017) and also an inhibitor of PI3K/AKT/mTOR signaling pathway (Adhami et al., 2012), which regulates key cellular processes including autophagy, and is discussed below in this article. In the context of inflammatory pulmonary diseases, Fisetin has demonstrated its therapeutic potential in murine models of allergic airway inflammation (Goh et al., 2012; Brown et al., 2016), and lipopolysaccharide (LPS) induced acute lung injury in rats, through its NFκB-targeted anti-inflammatory mechanism of action (Feng et al., 2016). Moreover, our recent report using cigarette smoke (CS)-extract and Pa model in murine macrophages, demonstrates the efficacy of Fisetin in correcting the CS-induced defect in bacterial clearance via transcription factor-EB (TFEB)-mediated autophagy-induction, and/or by restoring expression of mature (WT)-CFTR (Pehote et al., 2017). Additionally, similar to cysteamine, Fisetin also reveals direct bactericidal activity against Pa bacteria, a predominant CF-pathogen, by hitherto unknown mechanism(s) (Pehote et al., 2017). In another parallel study, using CS-exposure of retinal pigment epithelial cells (RPE) as an *in vitro* model of age-related macular degeneration (AMD), Fisetin successfully corrected the CS-induced autophagy-flux impairment and reduced the perinuclear accumulation of aggresome bodies, plausibly by controlling CS-induced ROS-activation (Govindaraju et al., 2017). Since CF lung disease is also characterized by chronic inflammatory-oxidative stress, persistent bacterial infections and autophagy impairment, proof of concept *in vitro* data warrants evaluation of the efficacy of Fisetin in pre-clinical CF-lung disease models. Although, it should be noted that similar to other promising autophagy-inducing drug candidates, the utility of Fisetin is hampered by its poor aqueous solubility (Bothiraja et al., 2014) and low oral bioavailability (Seguin et al., 2013), and thus attempts have been underway to improve its *in vivo* efficacy by the use of nanotechnology-based airway-delivery approaches (Ragelle et al., 2012; Kadari et al., 2017; Mehta et al., 2018).

## A Thymic Peptide to Correct the Basic CF-Defect

**FIGURE 3:**
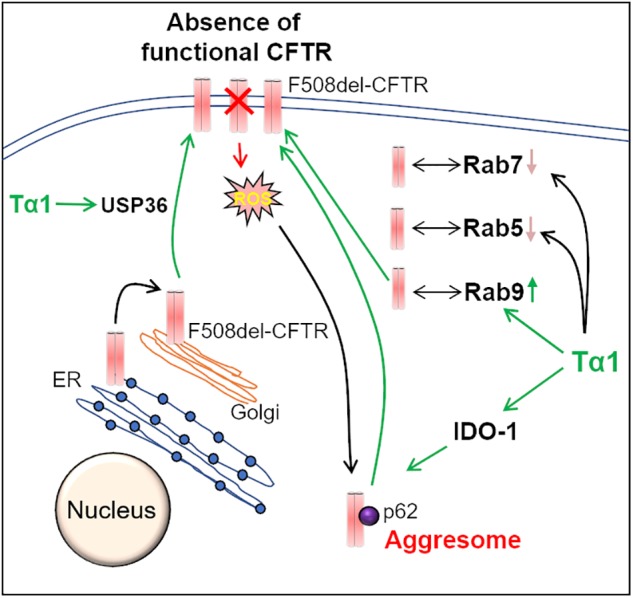
Thymosin-α-1 acts on multiple-targets to rescue F508del-CFTR. Thymosin α-1 (Tα1) is a thymic peptide, which acts on multiple cellular pathways in the CFTR recycling and maturation process to restore the misfolded F508del-CFTR to the plasma membrane (PM). First, Tα1 can induce autophagy via activation of indoleamine 2, 3-dioxygenase (IDO-1), and reduction of p62 levels, thereby resulting in the rescue of F508del-CFTR to the PM. Additionally, Tα1 activates deubiquitinating enzyme USP36, which in turn prevents the ubiquitination and subsequent degradation of F508del-CFTR, thus improving its PM stability. Moreover, in Tα1 treated cells, F508del-CFTR is found to be associated with Rab9 GTPase (recycling endosome marker), which promotes recycling of F508del-CFTR to the PM. This happens in parallel to a decrease in co-localization of F508del-CFTR with Rab 5 (early endosome marker) and Rab7 (late endosome marker) GTPases, in Tα1 treated cells.

Recent studies have highlighted the potential of Thymosin α-1 (Tα1), a thymic peptide with broad immune-modulatory properties, in correcting the basic CF-defect, i.e., the restoration of misfolded F508del-CFTR to the PM (Romani et al., 2017; Garaci, 2018; Rubin, 2018; Stincardini et al., 2018). Mechanistically, activation of indoleamine 2, 3-dioxygenase (IDO1), and the resulting decrease in inflammation, along with autophagy-induction are proposed as the key means of Tα1-mediated F508del-CFTR rescue (Romani et al., 2017) (Figure 3). Tα1 was shown to rescue F508del-CFTR to the PM at a clinically achievable dose, and this was attributed to its activity as a proteostasis modulator. Tα1 act’s on multiple steps of F508del-CFTR recycling such as the Rab GTPase’s, the deubiquitinating enzyme USP36, and the ubiquitin-binding protein, p62, which is involved in the aggresome sequestration of F508del-CFTR (Romani et al., 2017). In Tα1 treated cells, the F508del-CFTR co-localized with Rab9, which is the marker of recycling endosome. Moreover, the co-localization of F508del-CFTR with Rab5 (early endosome marker) and Rab7 (late endosome marker) was diminished by Tα1 treatment. Thus, Tα1 reduces the endocytic recycling of F508del-CFTR into early endosomes and also prevents its transport into late endosomes and/or lysosomes, thereby promoting its forward recycling to the PM (Romani et al., 2017). Although Tα1 has a good clinical safety profile and is already available commercially as ZADAXIN^®^ for the treatment of several inflammatory and/or infectious diseases, such as viral infections, immunodeficiency diseases, HIV/AIDS and cancers (Romani et al., 2017), it remains to be investigated whether it possesses other important anti-CF attributes such as bactericidal and mucolytic. Moreover, some recent studies report that Tα1 failed to rescue CFTR in epithelial cells and primary bronchial epithelial cells from CF patients (Matthes et al., 2018; Tomati et al., 2018), although these effects may be due to the incorrect solvent used by these investigators (Garaci, 2018). Thus, the recent claims that Tα1 could be a potential “single-molecule” drug for preventing/treating chronic CF-lung disease seems to be a far shot that requires further in-depth studies in pre-clinical and clinical CF settings.

## PI3K/AKT/mTOR Inhibitors as Emerging CF Drugs

The mammalian target of rapamycin (mTOR) is a member of the phosphatidylinositol 3-kinase (PI3K)-related kinase family of proteins that has long been implicated in regulating key cellular processes such as cell growth (Yu and Cui, 2016), survival (Yu and Cui, 2016), motility (Holroyd and Michie, 2018), metabolic pathways (Yu and Cui, 2016) and autophagy (Kim and Guan, 2015; Rabanal-Ruiz et al., 2017). The PI3K/AKT/mTOR signaling pathway is altered in several disease states such as cancer (Conciatori et al., 2018; Guri et al., 2018), immune system-related diseases (Guri et al., 2018; Jung et al., 2018), idiopathic pulmonary fibrosis (IPF) (Lawrence and Nho, 2018), COPD (Houssaini et al., 2018; Wang et al., 2018) and lymphangioleiomyomatosis (LAM) (Gao et al., 2018). Since mTOR is considered to be the master regulator of the autophagy pathway, its inhibitors have been investigated for their therapeutic potential in different types of cancers, and autophagy-induction is one of the proposed mechanisms of action (Saxton and Sabatini, 2017; Paquette et al., 2018). Moreover, in fibroblasts, higher than normal mTOR activity and the resulting autophagy-defect has been associated with pathogenesis of IPF (Lawrence and Nho, 2018), a fatal chronic restrictive lung disease. Additionally, elevated mTOR signaling mediated autophagy impairment was recently observed in lung cells and tissues isolated from COPD subjects, while the same conceptual evidence was also derived from transgenic mice with constitutive or conditional over-activation of mTOR (Houssaini et al., 2018). Interestingly, lung cell senescence and development of emphysema was found to be associated with elevated mTOR activity in these mice, as the effects could be ameliorated by rapamycin (an mTOR inhibitor) (Houssaini et al., 2018). It is also noteworthy that mTOR inhibition has been beneficial in the clearance of protein aggregates (aggresomes) in neurogenerative diseases (Heras-Sandoval et al., 2014), thus further confirming the crucial role of mTOR in regulating the autophagy process. Rapamycin mediated mTOR inhibition has been shown to reduce lung inflammatory responses in a CF mouse model (Abdulrahman et al., 2011), along with improved CFTR function (Luciani et al., 2011; Tazi and Amer, 2015). These studies confirmed that restoration of autophagy using Rapamycin, which is commercially available as Sirolimus, could benefit CF patients, although it has several limitations in clinical practice (Emoto et al., 2013; Li et al., 2014). Sirolimus has low oral bioavailability (Brasttström C. et al., 2000), poor water solubility (Kim et al., 2011), a huge pharmacokinetic variability among patients (Emoto et al., 2013), adverse side effects (Bee et al., 2018), and off-target effects (Arriola et al., 2016; Lamming, 2016; Haeri et al., 2017), which are due to its inhibition of both mTORC1 and mTORC2 (Arriola et al., 2016). The primary side effects of sirolimus include hyperglycemia, hyperlipidemia, insulin resistance and increase in new onset of type 2 diabetes (Emoto et al., 2013; Bee et al., 2018). In a national cohort study, the lung function response to rapamycin treatment and its associated side effects in women with progressive lung disease due to LAM was investigated (Bee et al., 2018). It was observed that although side effects were common, but they were manageable over several years, and improvements in lung function were evident. Overall, a low dose rapamycin was associated with fewer side effects with no difference in the beneficial effects (Bee et al., 2018), thus warranting its further clinical evaluation in CF. Moreover, several studies have been conducted to devise ways to enhance the bioavailability and improve *in vivo* delivery of sirolimus (Kim et al., 2011; Haeri et al., 2017). Considering the central role of impaired-autophagy and resulting aggresome-pathology in CF, it seems worthwhile to test the efficacy of mTOR inhibitors in CF models. In accord with this idea, CFBE41o- cells demonstrated upregulated mTOR activity, and the resulting autophagy impairment was found to be associated with accumulation of F508del-CFTR into peri-nuclear aggresome bodies (Reilly et al., 2017). Moreover, the inhibition of PI3K/AKT/mTOR pathway by six different compounds enhanced CFTR-membrane stability and expression (Reilly et al., 2017). The study identified MK-2206 as the most potent CFTR rescuing compound, which functions through targeting Bcl-2-associated athanogene 3 (BAG3), a regulator of autophagy and aggresome clearance (Reilly et al., 2017). Thus, the efficacy of pharmacological PI3K/AKT/mTOR inhibitors warrants further evaluation as potential therapeutic candidates for chronic CF lung disease, based on their ability to rectify the disease-promoting proteostasis and autophagy defect, including the correction of underlying CFTR dysfunction.

## HDAC Inhibitors as Proteostasis Modulators in CF

Inhibition of histone deacetylases (HDAC) has been evaluated as a potential therapeutic strategy for several protein folding and other chronic inflammatory diseases such as neurodegenerative diseases (Benito et al., 2015; Rabal et al., 2016), chronic kidney disease (Liu and Zhuang, 2015), inflammatory bowel disease (Felice et al., 2015), cancer (Falkenberg and Johnstone, 2014; West and Johnstone, 2014; De Souza and Chatterji, 2015), graft-versus-host disease (Choi et al., 2014, 2017), rheumatoid arthritis (Oh et al., 2017) and CF (Hutt et al., 2010, 2011; Bodas et al., 2018a). Pharmacological studies of suberanilohydroxamic acid (SAHA, Vorinostat), a broad inhibitor of class I and II HDAC enzymes (Bubna, 2015), in different types of cancers indicate that SAHA is well tolerated and demonstrates good oral bioavailability (43%) (Kelly et al., 2005). Moreover, the major adverse effects of SAHA administration such as fatigue, diarrhea, dehydration, etc., where more prominent in the intravenous treatment route, rather than the oral treatment regime, and the more severe indications such as thrombocytopenia, were resolved upon discontinuation of treatment (O’Connor et al., 2006; Bubna, 2015). Additionally, SAHA is also an FDA approved drug for cutaneous T-cell lymphoma (Bubna, 2015). Thus, at least in patient-based studies targeting cancer, SAHA was safely administered over a prolonged period, with minimal toxicity and consistent anti-HDAC activity, thereby indicating its potential tolerance as a CF drug candidate. In CF, the pharmacological inhibition of HDACs, especially using SAHA seems encouraging as this provides twofold benefit of controlling the inflammation (Hull et al., 2016; Xu et al., 2017) and also function as a proteostasis regulator (Bouchecareilh et al., 2012; Han et al., 2015) to facilitate rescue and trafficking of F508del-CFTR to the PM (CFTR-corrector) (Bodas et al., 2018a). Indeed, we recently verified the potential utility of SAHA in rescuing the F508del-CFTR to the PM by delaying its degradation, thus confirming its potential as a CFTR-corrector (Bodas et al., 2018a). Additionally, SAHA treatment was also effective in controlling *Pa*-LPS induced inflammation and neutrophil activation in a pre-clinical CF murine model, which was possibly *via* induction of regulatory T cells (Bodas et al., 2018a). Intriguingly, this observation was CFTR-independent, as the inflammation quenching function of SAHA was evident even in C*ftr^-/-^* mice. This indicates that SAHA could provide a potential therapeutic benefit in CF irrespective of its ability to rescue mutant CFTR. An ostensibly contrasting study demonstrates the failure of SAHA to restore F508del-CFTR, albeit the cells and the dose of SAHA used in those reports are dissimilar to our studies, which possibly explains the disparity in the results (Bergougnoux et al., 2017). Moreover, in two other studies SAHA was able to increase forskolin-induced chloride secretion in cell lines expressing CFTR but failed to demonstrate the same effect in primary epithelial cells from CF patients (Sondo et al., 2011; Van Goor et al., 2011). Nonetheless, proteostasis regulators such as SAHA and specific HDAC 6/7 inhibitors, such as tubacin (Cebotaru et al., 2008) have been evaluated in rescuing misfolded F508del-CFTR from proteasomal degradation and aggresome-accumulation. Further pre-clinical studies are necessary to evaluate the therapeutic efficacy of specific HDAC inhibitors, which might be coupled with novel drug-delivery systems (Mohamed et al., 2012; Tran et al., 2014) to further enhance their *in vivo* efficacy and bioavailability in CF lungs.

Briefly, as a proof of concept in support of proposed strategy adapting proteostasis and autophagy for rescuing the CF lung disease, recent study demonstrates that VX-809 mediated CFTR rescue is proteostasis-dependent but autophagy-independent (Pesce et al., 2018), where potent autophagy augmentation will allow synergistic effects on both mutant-CFTR rescue and *other components* (Romani et al., 2017; Stincardini et al., 2018) of CF lung disease pathogenesis as discussed in detail above (De Stefano et al., 2014; Tosco et al., 2016; Vu et al., 2017; Hutt et al., 2018; Stincardini et al., 2018; Zhang et al., 2018). In this study investigators, attempted to augment autophagy using torin-1 (Pesce et al., 2018) but its effects are missed due to lack of serum-starvation and appropriate experimental conditions. Nonetheless, extensive body of experimental evidence from our group and other’s suggest that adapting proteostasis and autophagy has significant potential in correcting the underlying causes of CF lung disease pathogenesis and will allow development of next generation of potent novel therapeutics as summarized below.

## Perspective

The absence of a functional membrane CFTR is the primary etiology of chronic lung disease development in CF patients, which progresses due to numerous pathological complications such as mucus-overproduction, elevated oxidative stress, chronic infections and sustained NFκB-mediated inflammation, eventually leading to early-life fatality, if left untreated. Although huge strides have been made in the development of novel “breakthrough” drug combinations such as Orkambi^®^, Symdeko^®^ etc., to rectify the core CF-defect, their widespread therapeutic advantage has been restricted due to somewhat low efficacy in maintaining sustained CFTR-activation as well as controlling other components of CF lung disease such as chronic inflammatory-oxidative stress responses and exacerbations. Therefore, alternative therapeutic methodologies using novel drugs and/or drug-delivery systems need to be concurrently developed, which can fill the gap of an affordable yet potent and effective CF treatment strategy capable of rescuing overall CF lung disease. Since, significant experimental and pre-clinical evidence suggests the key central role of proteostasis and autophagy processes in regulating most of the disease-causing pathogenic features in the CF airways, this warrants further clinical evaluation and development of proteostasis and autophagy modulating drugs, as an emerging therapeutic approach for CF lung disease. Finally, since CF subjects possesses numerous types of CFTR mutations, genotyping of the patient before deciding on the proteostasis and autophagy modulating drug(s), will allow evaluating the therapeutic advantage for the patient as a part of emerging Precision Medicine Initiative.

## Author Contributions

Both authors contributed to the concept, framework and writing of the manuscript for publication.

## Conflict of Interest Statement

NV is the lead inventor on patent targeting proteostasis mechanisms for rescuing CFTR protein-processing defect and CF lung disease and also a founder of VIJ Biotech that focuses on bench-side translation of novel CF and COPD therapeutics. The remaining author declares that the research was conducted in the absence of any commercial or financial relationships that could be construed as a potential conflict of interest.
